# Siglec-1 upregulation and macrophage activation in age-related hearing loss: insights from integrated transcriptomic studies in mice and humans

**DOI:** 10.3389/fncel.2026.1879068

**Published:** 2026-07-03

**Authors:** Di Ji, Qian Li, Baiyang Ren, Hongli Lan, Wenqi Zuo, Tao Chen, Xin Gong, Sen Yang, Anchun Deng, Shixun Zhong

**Affiliations:** 1 Department of Otolaryngology, The First Affiliated Hospital of Chongqing Medical University, Chongqing, China; 2 Department of Thoracic Surgery, The First Affiliated Hospital of Chongqing Medical University, Chongqing, China; 3 Department of Otolaryngology, The Second Affiliated Hospital of the Army Military Medical University, Chongqing, China; 4 Department of Otolaryngology, Suining Central Hospital, Suining, China

**Keywords:** age-related hearing loss, inflammatory cytokine, macrophage, neuroinflammation, Siglec-1, spiral ganglion

## Abstract

Macrophage activation is closely associated with age-related hearing loss (ARHL), and Siglec-1 may be involved in modulating macrophage activation. This study aimed to investigate the immunological mechanisms underlying spiral ganglion neuron (SGN) degeneration in ARHL, with a focus on the roles of the immune regulatory gene Siglec-1 and immune regulatory cells, macrophages. Combined transcriptomic sequencing of SGNs from ARHL mice and peripheral blood samples from ARHL patients identified significant upregulation of Siglec-1 in aged groups. Auditory brainstem response (ABR) testing and histological analysis confirmed that hearing thresholds increased and SGN density decreased significantly with age, exhibiting a strong negative correlation. Subsequently, both immunofluorescence and Western blot analyses revealed a notable upregulation of Siglec-1 expression within the spiral ganglion of aged mice. Further RT-PCR analysis revealed that the expression levels of both pro-inflammatory cytokines (including IL-6, TNF-*α*, and IFN-*γ*) and the anti-inflammatory cytokine IL-10 were significantly elevated in the aging spiral ganglion tissue. Meanwhile, notable macrophage activation was observed within the spiral ganglion, particularly involving M1 pro-inflammatory macrophages. These findings suggest that Siglec-1 may play a role in the inflammatory response. Finally, we also confirmed a positive correlation between Siglec-1 mRNA expression in human blood samples with age and severity of hearing loss. These findings suggest that Siglec-1 may promote SGN degeneration in ARHL by modulating macrophage-mediated neuroinflammation and represents a potential blood immune biomarker and therapeutic target.

## 1 Introduction

Age-related hearing loss (ARHL) is a prevalent sensory disorder characterized by progressive deterioration in auditory function, significantly impacting the quality of life in the aging population (Ege et al., 2024; Haile et al., 2024). A key pathological feature of ARHL is the degeneration of spiral ganglion neurons (SGNs), which play a crucial role in transmitting auditory signals from the cochlea to the brain. Despite extensive research, the molecular mechanisms underlying SGN degeneration during aging remain incompletely understood.

Emerging evidence suggests that immune-related processes contribute to neurodegeneration in various sensory systems (Kaur et al., 2015; Watson et al., 2017; Keithley, 2020), prompting investigation into immunological changes within the aging cochlea. Macrophages are the primary effector cells of cochlear immunity and play a crucial role in maintaining inner ear homeostasis and repairing damage. They are distributed across multiple anatomical sites, primarily including the spiral ganglion and stria vascularis (Lim, 1970; Okano et al., 2008; Sato et al., 2010; Hirose and Li, 2019). Recent studies have highlighted the involvement of pro-inflammatory cytokines, such as tumor necrosis factor-alpha (TNF-*α*) and interleukin-1β (IL-1β), in the aging cochlea (Fuentes-Santamaría et al., 2022). The elevated levels of these cytokines are associated with increased oxidative stress (Zhao et al., 2022) and neuronal apoptosis (Choi et al., 2016; Nguyen et al., 2022; An et al., 2026), thereby exacerbating age-related hearing decline. Notably, experimental evidence demonstrates that inhibition of spiral ganglion neuron apoptosis can attenuate age-related hearing loss. Furthermore, the activation of the NLRP3 inflammasome, a key component of the innate immune system, has been implicated in cochlear inflammation and degeneration (Zheng et al., 2024), suggesting a potential target for therapeutic intervention.

Despite these insights, the precise mechanisms by which immune-related processes influence spiral ganglion aging and neurodegeneration remain inadequately understood. Current research is limited by a lack of comprehensive models that accurately replicate the immunological environment of the aging spiral ganglion. Additionally, the interplay between systemic inflammation and local spiral ganglion immune responses warrants further exploration to elucidate their contributions to auditory pathologies, particularly in the context of age-related hearing loss and other auditory disorders.

The stable expression of peripheral blood inflammatory markers (e.g., C-reactive protein, IL-6, IL-1β and TNF-*α*) reflects systemic inner ear inflammation, offering a practical and non-invasive monitoring approach (Fujioka et al., 2006; Verschuur et al., 2012). Validating these inflammatory markers within the spiral ganglion would establish their clinical significance for non-invasive, liquid biopsy-based early warning of ARHL. Dynamic tracking of these factors enables early detection of hearing loss risk in the elderly, supporting timely intervention. Consistent inflammatory markers enhance early warning accuracy and sensitivity, reduce misdiagnosis, and advance personalized medicine.

Therefore, we conducted a combined transcriptomic analysis of spiral ganglion tissues from ARHL mice and blood samples from ARHL patients, identifying Siglec-1. Our transcriptomic analyses have identified Siglec-1, an immune regulatory gene, as significantly upregulated in aged SGNs and peripheral blood, implicating it as a potential mediator of age-associated spiral ganglion pathology. Moreover, the increased expression of Siglec-1 is associated with macrophage activation within the spiral ganglion and elevated levels of pro-inflammatory cytokines, suggesting that the inflammatory environment may exacerbate neuronal loss (Table 1).

**Table 1:** Sequences of primers used in this study.

Description	Orientation	Sequence (5′-3′)
IL-1β	Forward	GCCTGTGTTTTCCTCCTTGC
	Reverse	TGCTGCCTAATGTCCCCTTG
IL-6	Forward	GCCTAAGCATATCAGTTTGTGGA
	Reverse	CCAACATTCATATTGTCAGTTCTTCG
IL-10	Forward	TGATGGGAGGGGTTCTTCCT
	Reverse	GGGATGACAGTAGGGGAACC
TNF-α	Forward	AACCCAATTGTCTTAATAACGCTG
	Reverse	ATGACCCGTAGGGCGATTAC
IFN-γ	Forward	TGTGTCAGGTAGTAACAGGCT
	Reverse	TGTCATTCGGGTGTAGTCACAG
GAPDH (mice)	Forward	ACTCTTCCACCTTCGATGCC
	Reverse	TGGGATAGGGCCTCTCTTGC
GAPDH (humans)	Forward	GTCTCCTCTGACTTCAACAGCG
	Reverse	ACCACCCTGTTGCTGTAGCCAA
Siglec-1	Forward	ACCTGGAGGAAACTGACAGTGG
	Reverse	CTCAGTGTCACTGCCTGTCCTT

This study aims to elucidate the relationship between ARHL and SGN degeneration, focusing on the role of Siglec-1 and associated immune responses in SGN aging. By integrating functional auditory assessments, histological analysis, and molecular profiling, we seek to clarify the contribution of immune-mediated mechanisms to SGN loss and identify novel targets for therapeutic intervention in age-related auditory decline.

## 2 Materials and methods

### 2.1 Animals

All experimental animal protocols were performed following the Laboratory Animal Welfare and Ethics Committee OF the Army Medical University (Approval Letter No. AMUWEC20265508). 3-, 5-, 8-, 12-, 18-month-old male C57BL/6 J mice used in this study were purchased from ZhiShan (Beijing) Health Medicine Research Institute Co., Ltd. and kept in a quiet environment with enough water and food under a standard 12 h: 12 h light–dark cycle.

### 2.2 Patients

Patients who presented to the Department of Otolaryngology at The Second Affiliated Hospital of Army Medical University from April 2025 to May 2025 and met the inclusion criteria were selected and assessed for eligibility by an otolaryngologist. This study was approved by the Institutional Ethics Committee (Medical Ethics Committee of Second Affiliated Hospital of Army Medical University) (2025-Research No. 125–01). Song Caiping is the Principal Investigator of the Institutional Ethics Committee. The research was conducted ethically, with all study procedures being performed in accordance with the requirements of the World Medical Association’s Declaration of Helsinki (JAMA 2000; 284: 3043–3,049). Written informed consent was obtained from each participant/patient for study participation and data publication. The inclusion criteria for the young adult normal hearing group were defined as individuals under 35 years of age with no significant medical conditions and normal pure-tone audiometry results. The inclusion criteria for the elderly group with age-related hearing loss were defined as individuals aged 65 years and above, with hearing loss caused by age rather than by any disease.

### 2.3 ABR testing

ABR measurements were performed as previous described(Li et al., 2022). Briefly, mice were anesthetized with ketamine (100 mg/kg) and xylazine (10 mg/kg). Three silver wire electrodes were inserted subcutaneously at the vertex of the skull, the mastoid process of the tested ear (recording) and the mastoid process of contralateral ear (ground). ABR was measured using a Smart-EP Auditory Evoked Potential Instrument (Intelligent hearing Systems, USA). Responses were measured for clicks and pure tones at various frequencies. The hearing threshold was determined as the lowest stimulus level at which a repeatable Wave II could be visually identified.

### 2.4 SGNs morphometry and counting

The cochleae were fixed in 4% paraformaldehyde and decalcified in 10%.

EDTA at 4 °C. The decalcified cochleae were dehydrated by gradient alcohol and then embedded in paraffin, and cut into 5 μm sections. To evaluate SGNs morphometry and density, cochlear sections were stained with hematoxylin and eosin. Images were captured using an Upright optical microscope (NIKON Japan, NIKON ECLIPSE E100, Tokyo, Japan). Three cochlear samples from each subgroup were used for SGNs counting. The Rosenthal’s canal was divided into three regions: the apex, middle, and base. The number of SGNs in each section was divided by the area of Rosenthal’s canal, and the total number of SGNs was counted using Image J software. Finally, the density of SGNs was calculated within the unit area (10,000 μm^2^) from each section of cochlea.

### 2.5 Immunofluorescence

The antibody concentration was anti-CD169 (1: 2000, Affinity, DF13669, Waltham, Beijing, China), anti-F4/80 (1: 5000, servicebio, GB113373, Wuhan, Hubei), anti-Tuj1 (1: 3000, servicebio, GB15139, Wuhan, Hubei), anti-MHC-II (1: 400, Affinity, DF6475, Waltham, Beijing, China), anti-CD206 (1: 1000, Huaan, HA722892, Xiamen, Fujian). Images were obtained with a Upright fluorescence microscope (Nikon, Nikon Eclipse C1, Tokyo, Japan).

When calculating fluorescence intensity and cell density, we randomly selected samples and identified the anatomical landmark of the spiral ganglion—Rosenthal’s canal—under a microscope. Subsequently, we magnified the images to the same degree and captured photographs with identical shape and size, ensuring that the field of view encompassed the largest possible area of spiral ganglion tissue within Rosenthal’s canal while excluding non-target tissues from surrounding regions, such as the cochlear shell and basilar membrane, to minimize interference. Average fluorescence intensity and cell density were then quantitatively analyzed using ImageJ software under consistent parameters, followed by statistical analysis conducted with Xiantao Academic Software. For clear and precise structural localization, the photographs presented to showcase the immunofluorescence results included portions of the tissue surrounding Rosenthal’s canal. Additionally, we performed fluorescence localization of TUJ1, a specific marker for the spiral ganglion.

### 2.6 Quantitative real-time PCR

The total RNA sample from mouse spiral ganglion (8 mice were used for each sample) was extracted at 4 °C using Trelief ® RNAprep FastPure Tissue and Cell Kit (Cat: TSP413, Tsingke, Beijing, China) according to the established procedures. As in thereverse-transcription kit manual (Cat: TSK314S, Tsingke, Beijing, China), total RNA (~ 400 ng) were synthesized to cDNA using the SynScript ® III RT SuperMix for qPCR. Quantitative amplification was performed in triplicate on a QuantStudio™ 1 Plus Real-Time PCR System. The relative gene expression was analyzed by calculating the respective GAPDH using the formula: 2^-△△Ct.

Total RNA was extracted from the venous blood of 2 groups of 4 human individuals each at 4 °C. As in thereverse-transcription kit manual (JR0070, Jingrui Biology, Chongqing, China), total RNA (~ 200 ng) were synthesized to cDNA using the SynScript ® III RT SuperMix and SYBRPrime qPCR Set (JR0014, Jingrui Biology, Chongqing, China)for qPCR. Quantitative amplification was performed in triplicate on a QuantStudio™ 1 Plus Real-Time PCR System. The relative gene expression was analyzed by calculating the respective GAPDH using the formula: 2^-△△Ct.

### 2.7 Western blot

The proteins from mouse spiral ganglion tissues (3 mice were used for each sample) were extracted with strong RIPA lysis buffer containing.

PMSF (Youpin Biotechnology, YPW0017, Guangzhou, China). Then the protein concentration of each sample was measured by BCA kit following manufacturer’s instruction. Equal amounts of protein (30 μg) were separated using 10% or 12% SDS-PAGE gels (Youpin Biotechnology, YPW0001YPW0010, Guangzhou, China)depending on the molecular weight and then blotted onto PVDF membranes. After being blocked, the membranes were incubated overnight at 4 °C with a 1: 1000 dilution of Sialoadhesin Antibody (Affinity, DF13669, Jiangsu, China), and 1: 100000 dilution of *β*-actin (Sanying Biotechnology, 81,115-1-RR, Wuhan, China). Next day, the membranes were incubated with the secondary antibodies conjugated with horseradish peroxidase. At last, the visualization and data processing were performed by a ChemiScope Capture system. All experiments were repeated at least three times.

### 2.8 Transcriptome sequencing

Transcriptome sequencing was performed by Shenzhen Hepuross Biotechnology Co., Ltd. (Shenzhen, China) following standard procedures. In brief, after obtaining the experimental samples, mRNA is extracted and RNA sequence information is obtained through second-generation sequencing for differential gene analysis and functional enrichment analysis.

The specimens derived from mice for mRNA sequencing are spiral ganglion tissues. The specific method for obtaining these specimens is as follows: Under a microscope, other tissues such as the cochlear shell, basilar membrane, and bony tissue are carefully removed, leaving only the central white modiolus as the analytical specimen.

The quality control process is accomplished using the fastp software (version 0.23.0). The specific steps include: Adapter Sequence Removal: All adapter sequences are removed from the reads to prevent interference with subsequent analyses. Low-Quality Base Filtering: Reads containing ambiguous bases N (exceeding 5 bp by default) and reads with a high proportion (default 40%) of low-quality bases (quality value ≤ 20) are filtered out to ensure data accuracy. Sliding Window Quality Trimming: A sliding window (default size 4 bp) approach is employed to trim reads based on their average quality values. If the average quality within the window falls below the threshold (default 20), reads are trimmed from that point onwards to further enhance data quality.

The data analysis pipeline is as follows: Data Preprocessing and QC: Initial QC is performed using fastp to ensure data quality. Reference Genome Alignment: The cleaned reads are aligned to the reference genome using HISAT2 (version 2.1.0). Gene Expression Quantification: HTSeq (version 0.10.0) is used to quantify gene expression levels. Differential Gene Expression Analysis: DESeq2 (version 1.18.1) and edgeR (version 3.20.9) are employed to identify differentially expressed genes between sample groups.

GO Enrichment Analysis: Using the ClusterProfiler software package and based on the GO database, enrichment analysis is performed on differentially expressed genes across three categories: Molecular Function, Biological Process, and Cellular Component. By employing the hypergeometric distribution algorithm, the degree of enrichment of differentially expressed genes in each GO term is calculated, thereby revealing their potential roles in biological functions and processes.

The selection of immune-related genes and the choice of databases were primarily based on two aspects: literature review and database annotations. The ImmPort and KEGG databases were chosen to screen for genes closely associated with immune responses, and their expression patterns were visualized in a heatmap.

### 2.9 Statistical analysis

The data were presented as the mean ± SEM. Student’s t-test and Mann–Whitney nonparametric tests were performed for comparisons between the two groups. For comparisons more than two groups, statistical analysis was performed using one-way analysis of variance (ANOVA) followed by Tukey HSD *post hoc* test. Spearman’s test was performed to assess the correlation between two sets of data. A *p* value < 0.05 was considered statistically significant.

## 3 Results

### 3.1 Age-related hearing loss is associated with SGNs degeneration

**Figure 1:**
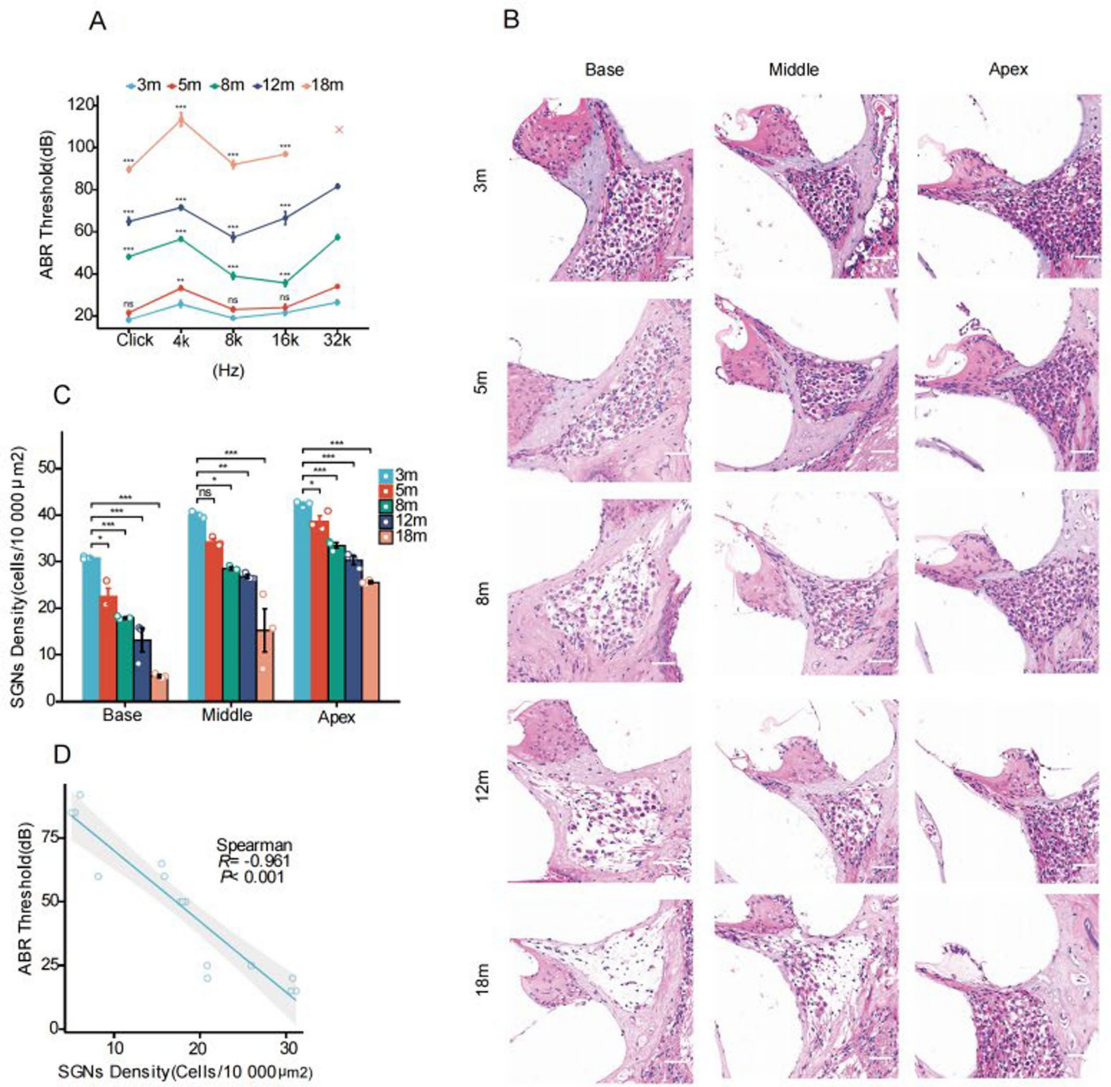
Age-related hearing loss and spiral ganglion neuron degeneration in the cochlea. **(A)** ABR thresholds at click and pure tone frequencies (4, 8, 16, 32 kHz) across 3, 5, 8, 12, and 18 months, showing significant age-related increases, especially at 18 months (**p < 0.01, ***p < 0.001, *n* = 6). **(B)** Representative hematoxylin and eosin-stained cochlear sections showing SGNs in basal, middle, and apical turns at different ages. **(C)** SGN density (cells/10,000 μm^2^) quantification across cochlear turns, revealing significant decreases with age (*p < 0.05, **p < 0.01, ***p < 0.001, *n* = 3). **(D)** Correlation between ABR thresholds and SGN density, demonstrating a strong inverse relationship (Spearman’s *R* = −0.961, *p* < 0.001). Scale bars in (**B**) represent 100 μm. Data are mean ± SEM. Statistical significance was assessed with one-way analysis of variance (ANOVA) followed by Tukey HSD *post hoc* test.

Auditory brainstem response (ABR) thresholds were measured across different frequencies (click, 4 k, 8 k, 16 k, and 32 k Hz) at multiple time points (3, 5, 8, 12, and 18 months). ABR thresholds increased significantly over time, with the 18-month group showing the highest thresholds, indicating progressive hearing loss (Figure 1A). Histological analysis of cochlear sections revealed a gradual loss of spiral ganglion neurons (SGNs) from the base to the apex regions with age (Figure 1B). Quantification of SGN density confirmed a significant decrease in cell numbers at all SGN turns as age progressed, with the most pronounced loss observed at 18 months (Figure 1C). A strong negative correlation was observed between ABR click stimulation thresholds and basal turn SGN density (Spearman *R* = −0.961, *p* < 0.001), indicating that higher hearing thresholds correspond to lower SGN density (Figure 1D).

### 3.2 Identification of Siglec-1 as a potential key gene in ARHL through transcriptomic analysis

**Figure 2:**
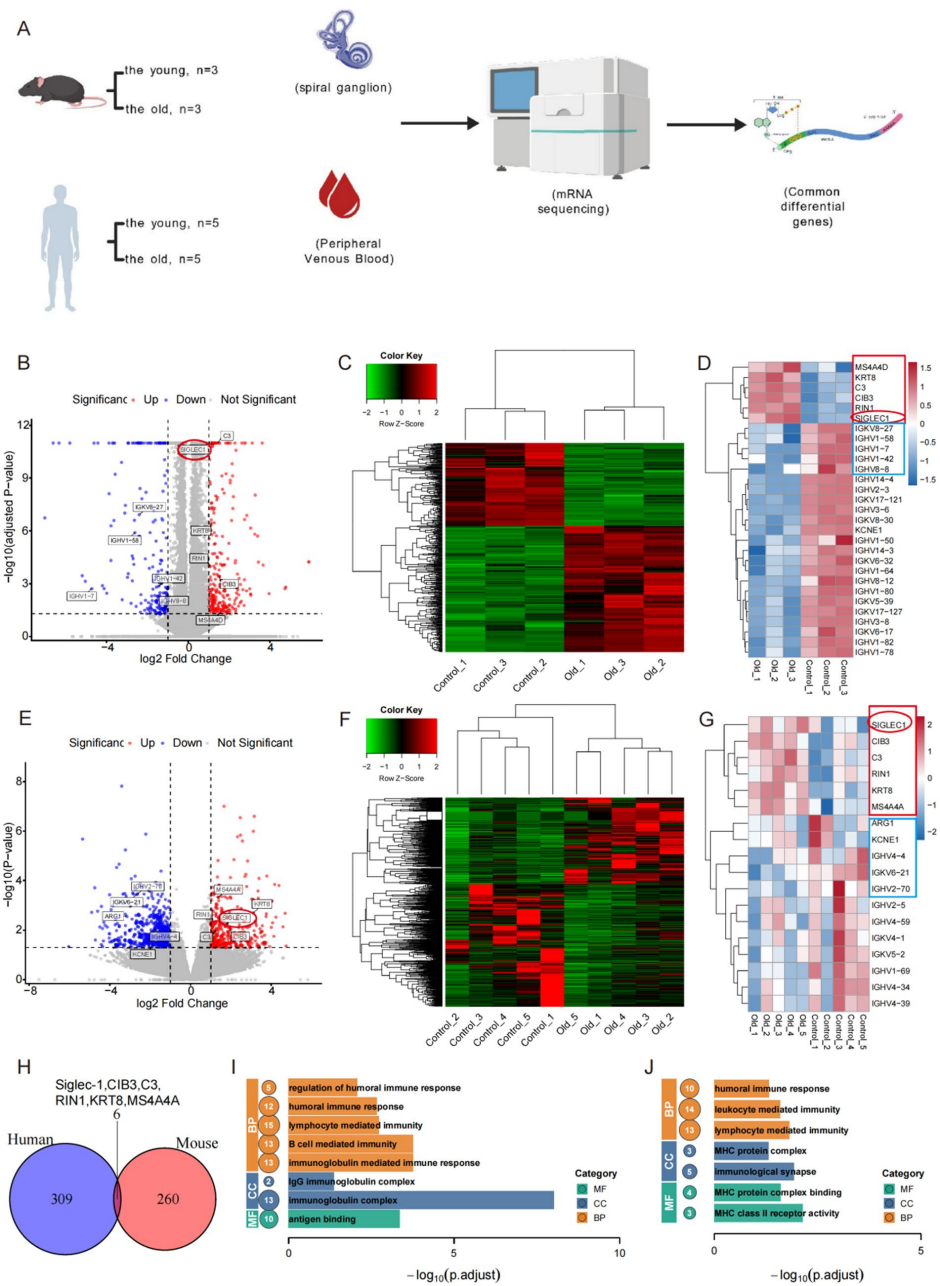
Identification and characterization of common aging-associated differentially expressed genes in mouse spiral ganglion neurons and human peripheral blood. **(A)** Schematic representation of the experimental design depicting mRNA sequencing performed on SGNs from young and aged mice and peripheral venous blood from young and aged humans to identify shared DEGs. (**B,E**) Volcano plots illustrate significantly upregulated (red) and downregulated (blue) genes in mouse SGNs **(B)** and human blood samples (**E**), with thresholds for statistical significance indicated. (**C,F**) Hierarchical clustering heatmaps of DEGs in mouse SGNs **(C)** and human blood **(F)** reveal distinct expression patterns between young and old groups. Color keys represent row Z-scores. (**D,G**) Heatmaps of selected immune-related genes, including Siglec-1, demonstrate consistent differential expression in aged samples of mouse SGNs (**D**) and human blood **(G)**. **(H)** A Venn diagram depicts the overlap of six common upregulated DEGs identified between mouse SGNs and human blood samples. (**I,J**) Gene Ontology enrichment analyses of common upregulated DEGs highlight significant biological processes related to immune response: **(I)** Enrichment analysis of biological functions in mice; **(J)** Human biological function enrichment analysis. Categories: BP, biological process; CC, cellular component; MF, molecular function. DEGs were visualized using volcano constructed based on stringent thresholds: an absolute log2-transformed fold change >1 and a Benjamini-Hochberg adjusted *p*-value <0.05 (mice) and p-value <0.05 (humans). Values exceeding 11 are uniformly marked as 11.

To identify common differentially expressed genes (DEGs) associated with aging in both mouse spiral ganglion neurons (SGNs) and human peripheral venous blood, mRNA sequencing was conducted on samples from young and old cohorts (mouse: *n* = 3 per group; human: *n* = 5 per group) (Figure 2A). Young individuals refer to those under the age of 35, while the elderly refer to the population aged 65 and above. For the mouse model, aged mice are selected at 12 months of age, and young mice are selected at 3 months of age. Volcano plot analyses revealed significantly upregulated and downregulated genes in mouse SGNs (Figure 2B) and human blood samples (Figure 2E). Hierarchical clustering heatmaps demonstrated distinct gene expression profiles differentiating young and old groups in both species (Figures 2C,F). Heatmaps focusing on selected immune-related genes highlighted consistent differential expression patterns, including the notable upregulation of Siglec-1 in aged samples (Figures 2D,G). Comparative analysis revealed six commonly upregulated DEGs between SGNs in mice and human peripheral blood, namely Siglec-1, Cib3, C3, Rin1, Krt8, and Ms4a4a (Figure 2H). Gene Ontology (GO) enrichment analysis of these shared DEGs emphasized their significant involvement in immune-related biological processes, such as regulation of humoral immune response, lymphocyte-mediated immunity, and immunoglobulin complex formation (Figures 2I,J).

### 3.3 Siglec-1 expression is upregulated in the spiral ganglion of aged mice

**Figure 3:**
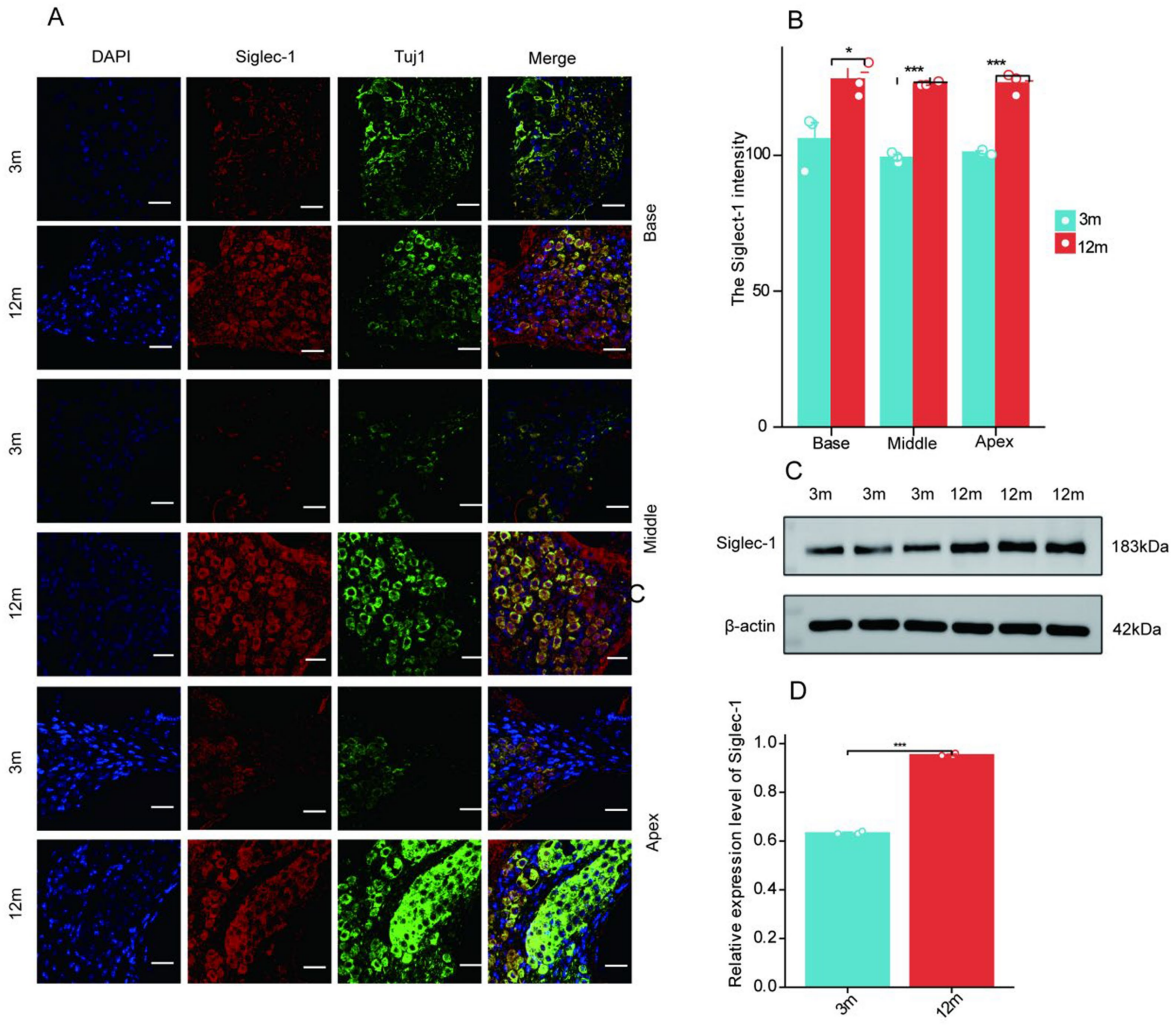
Age-associated upregulation of Siglec-1 in cochlear spiral ganglion neurons. **(A)** Representative immunofluorescence micrographs of cochlear spiral ganglion sections from 3-month-old (3 m) and 12-month-old (12 m) mice. Sections were stained with DAPI (blue) to visualize nuclei, Siglec-1 (red), and Tuj1 (green) to identify neuronal populations. Merged images illustrate the colocalization of Siglec-1 and Tuj1. Images correspond to the base, middle, and apex regions of the cochlea. Scale bars denote 50 μm. **(B)** Quantitative analysis of Siglec-1 fluorescence intensity within the base, middle, and apex regions of cochlear spiral ganglion neurons. Data are expressed as mean ± SEM, with individual data points indicated (*n* = 3). Statistical significance was evaluated using Student’s t-test (*p < 0.05, **p < 0.001). **(C)** Western blot analysis depicting Siglec-1 protein levels in SGN tissue lysates from 3-month-old and 12-month-old mice (*n* = 3). *β*-actin served as the loading control. Bands correspond to Siglec-1 (~ 183 kDa) and β-actin (~ 42 kDa).**(D)** Relative protein expression levels of Siglec-1 from 3-month-old and 12-month-old mice, normalized to control. Data are expressed as mean ± SEM, with individual data points indicated (*n* = 3). Statistical significance was evaluated using Student’s t-test (***p < 0.0001).

Age-Dependent Elevation of Siglec-1 Expression in Cochlear Spiral Ganglion Neurons Immunofluorescence analyses demonstrated a pronounced increase in Siglec-1 expression within spiral ganglion neurons of 12-month-old mice compared to those aged 3 months, consistently observed across all cochlear regions examined (base, middle, and apex). Co-localization with Tuj1 confirmed the neuronal specificity of Siglec-1-positive cells. Quantitative assessment of fluorescence intensity revealed a statistically significant augmentation of Siglec-1 levels in 12-month-old mice relative to 3-month-old controls in each cochlear region (Base: *p < 0.05; Middle and Apex: **p < 0.001) (Figures 3A,B). These findings were further supported by Western blot analysis, which showed elevated Siglec-1 protein expression in spiral ganglion tissue lysates from 12-month-old mice compared to 3-month-old counterparts (Figures 3C,D).

### 3.4 The upregulation of Siglec-1 may be associated with neuroinflammation mediated by macrophages and inflammatory cytokines

**Figure 4:**
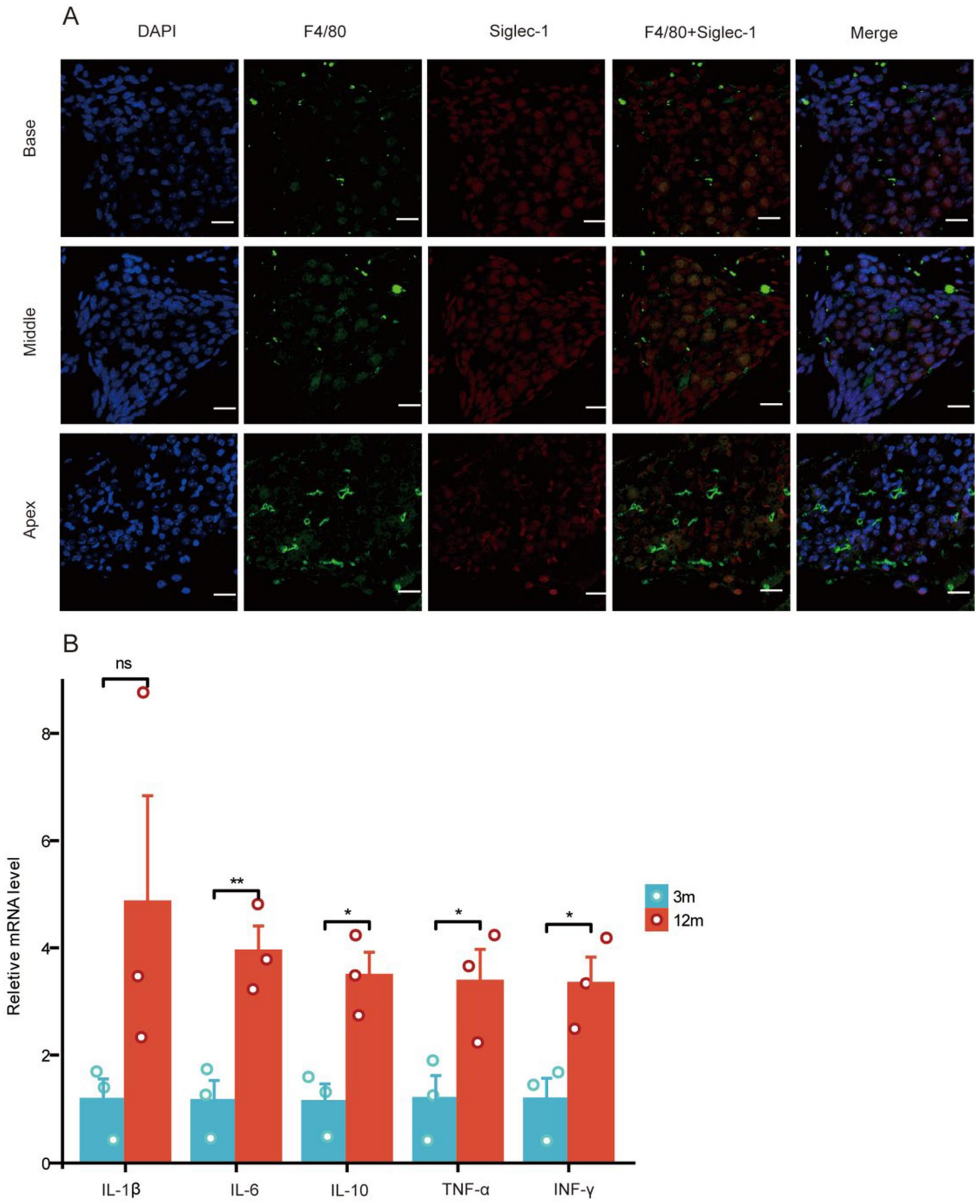
The inflammatory microenvironment of aging spiral ganglion neurons. **(A)** Representative immunofluorescence images of spiral ganglion neuron sections from mouse cochleae, stained with DAPI (blue), Siglec-1 (red), and the macrophage marker F4/80 (green). The images correspond to the basal, middle, and apex regions of the cochlea, respectively. Scale bar: 50 μm. **(B)** Relative mRNA expression levels of inflammatory cytokines IL-1β, IL-6, IL-10, TNF-*α*, and IFN-*γ* in SGN tissues from 3-month-old and 12-month-old mice, normalized to control. Data are presented as mean ± SEM (*n* = 3). Statistical significance was evaluated by Student’s t-test (*p < 0.05; **p < 0.001; ns, not significant).

To explore the immunological context of aging SGN, focusing on macrophage activation and inflammatory response linked to Siglec-1 expression. The results revealed a substantial overlap between Siglec-1-positive areas and F4/80-positive macrophage distribution, indicative of an inflammatory milieu (Figure 4A). Furthermore, quantitative RT-PCR analysis demonstrated significantly elevated mRNA expression levels of the pro-inflammatory cytokines IL-6, TNF-*α*, and IFN-*γ* in SGN tissues of 12-month-old mice compared to 3-month-old control mice, reflecting an age-related pro-inflammatory state. Concurrently, the anti-inflammatory cytokine interleukin-10 (IL-10) was also upregulated, suggesting the presence of a considerably complex inflammatory balance mechanism (Figure 4B).

### 3.5 Concomitant with increased Siglec-1 expression, age-related macrophages undergo activation, with a predominance of the M1 phenotype

**Figure 5:**
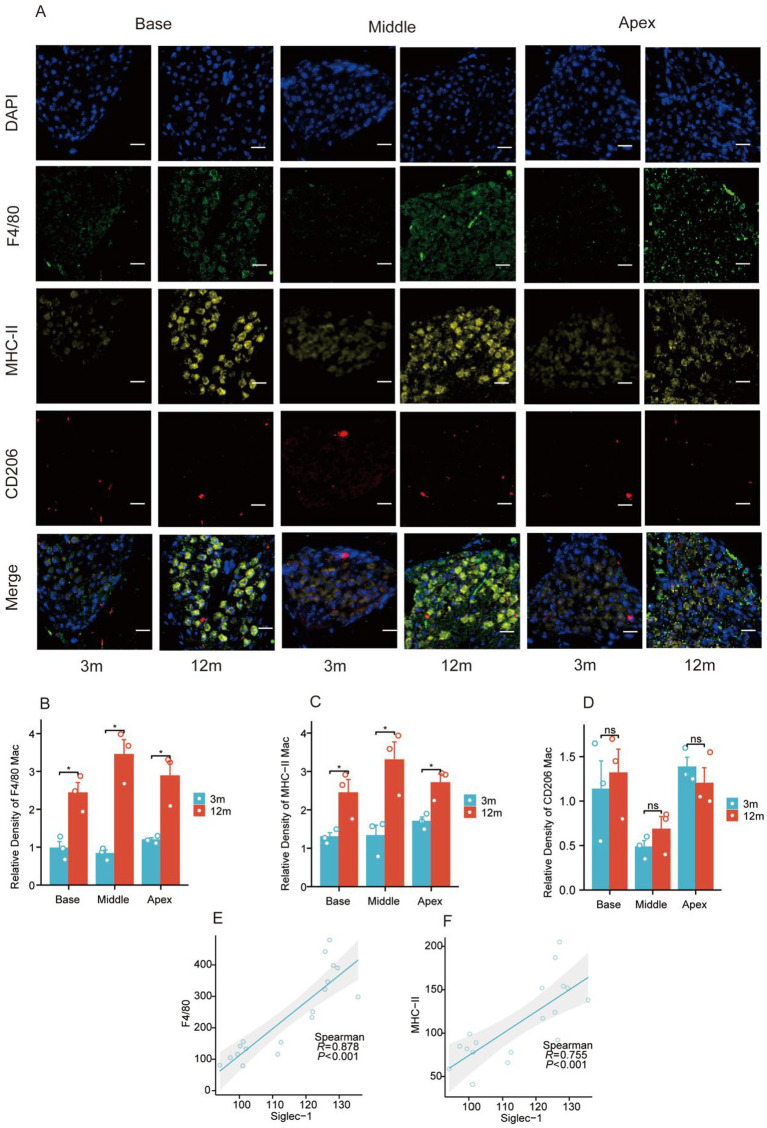
Activation status of age-related macrophages and its correlation with Siglec-1. **(A)** Representative immunofluorescence images of spiral ganglion sections from mouse cochleae, stained with DAPI (blue), the pan-macrophage marker F4/80 (green), the M1 macrophage activation marker MHC-II (yellow), and the M2 macrophage activation marker CD206 (red). The images correspond to the basal, middle, and apex regions of the cochlea, respectively. Scale bar: 50 μm. **(B)** The relative density of F4/80-positive macrophages in the spiral ganglion of 3-month-old and 12-month-old mice, normalized to the control group. **(C)** The relative density of MHC-II-positive macrophages in the spiral ganglion of 3-month-old and 12-month-old mice, normalized to the control group. **(D)** The relative density of CD206-positive macrophages in the spiral ganglion of 3-month-old and 12-month-old mice, normalized to the control group. Data are presented as mean ± SEM (*n* = 3). Statistical significance was assessed by Student’s t-test (*p < 0.05, ns = not significant). **(E)** The density of F4/80-positive macrophages was significantly positively correlated with Siglec-1 fluorescence intensity (Spearman’s *R* = 0.878, *p* < 0.001). **(F)** The density of MHC-II-positive macrophages was significantly positively correlated with Siglec-1 fluorescence intensity (Spearman’s *R* = 0.755, *p* < 0.001).

To further elucidate the activation status of age-associated macrophages, we performed immunofluorescence staining not only for the pan-macrophage marker F4/80 but also for M1 (MHC-II) and M2 (CD206) macrophages, respectively (Figure 5A). The results revealed that the density of F4/80-positive macrophages in the spiral ganglion of aged C57 mice (12 months) was overall higher compared to young mice (3 months) (*p* < 0.05) (Figure 5B). The density of MHC-II-positive macrophages was also significantly elevated (*p* < 0.05) (Figure 5C), whereas the density of CD206-positive macrophages showed no apparent change (*p* > 0.05) (Figure 5D). These findings indicate that with advancing age, macrophages progressively accumulate and become activated, with the activated phenotype being predominantly pro-inflammatory M1. Subsequently, we performed correlation analyses between Siglec-1 fluorescence intensity and the density of F4/80-positive macrophages as well as MHC-II-positive macrophages, respectively. Both analyses revealed strong positive correlations (F4/80: *R* = 0.878; MHC-II: *R* = 0.755) (Figures 5E,F).

### 3.6 mRNA expression of Siglec-1 is elevated in elderly patients and positively correlated with hearing levels

**Figure 6:**
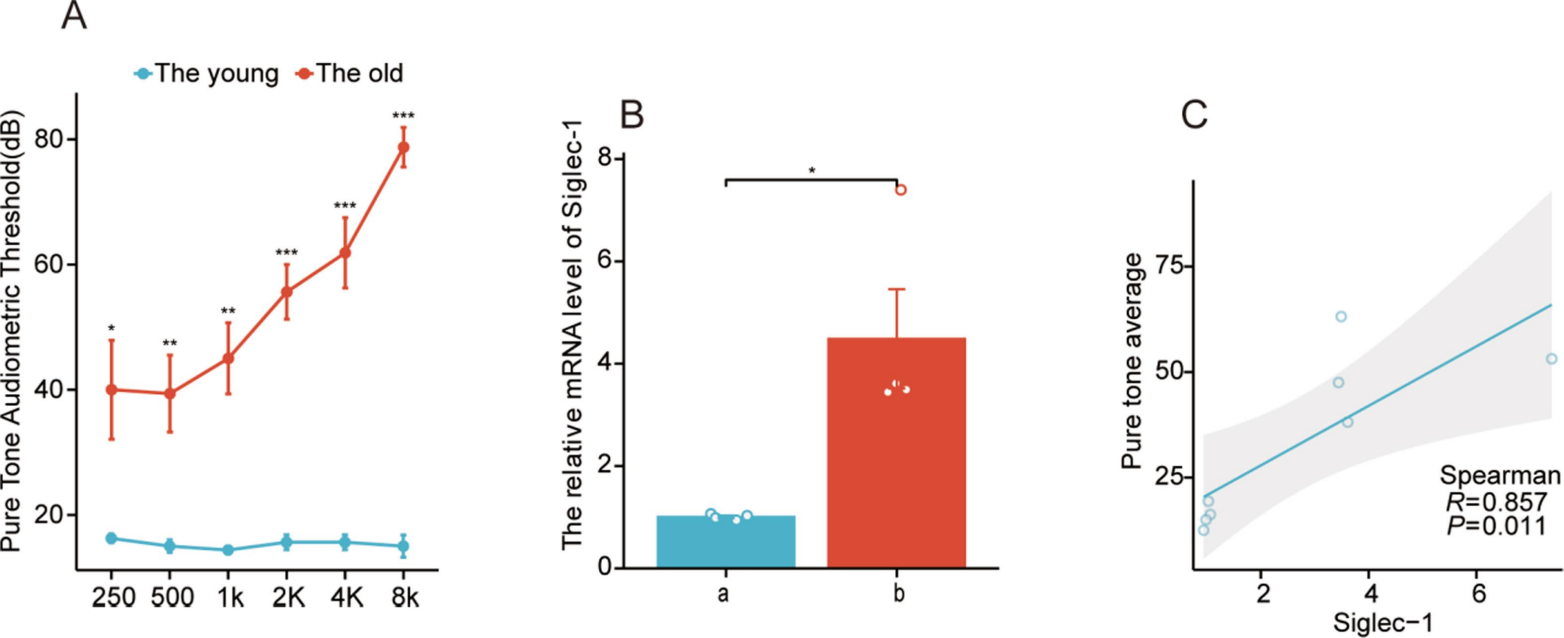
The expression of Siglec-1 mRNA in the elderly increases and is positively correlated with the hearing threshold. **(A)** Pure tone audiometry results of the old with hearing loss and the young with normal hearing (*n* = 4). **(B)** The expression of Siglec-1 mRNA in the venous blood of the two groups (*n* = 4). Statistical significance was determined by Student’s t-test (*p < 0.05). **(C)** Correlation analysis between the expression of Siglec-1 mRNA and Average pure tone audiometric threshold (Spearman’s correlation coefficient *r* = 0.857, *p* = 0.011).

To verify whether Siglec-1 has the same effect in the human body, we used quantitative RT-PCR analysis to detect the expression of Siglec-1 mRNA in the blood of healthy young people and elderly patients with deafness (Figure 6A). The results showed that the expression level in elderly patients with deafness was significantly higher than that in the other group (Figure 6B). Subsequently, we conducted a correlation analysis between the pure tone threshold average and the expression level of Siglec-1 mRNA. As expected, correlation analysis revealed a significant positive association between Siglec-1 mRNA expression levels and pure-tone hearing thresholds (Spearman’s *R* = 0.857, *p* < 0.05), indicating that Siglec-1 expression in blood increases with the severity of hearing loss (Figure 6C). These findings support the potential of Siglec-1 as a blood-based biomarker for age-related hearing loss, reflecting systemic immune-inflammatory status linked to auditory function decline.

## 4 Discussion

The present study highlights the key role of Siglec-1 in ARHL progression by regulating immune responses within the cochlear spiral ganglion. Its upregulation in aged SGNs and blood is linked to increased macrophage activation and disrupted inflammatory cytokine balance, fostering chronic inflammation that accelerates neuronal degeneration in the aging SGN.

Inflammatory factors such as TNF-*α* and IL-1β can directly act on receptors on nerve cells, activating intracellular signaling pathways and leading to cell apoptosis or necrosis. For instance, TNF-α binds to TNF receptors on the cell surface, activating the caspase protease family and triggering cell apoptosis. IL-1β can induce astrocytes and microglia to produce inflammatory mediators, including NO, ROS, and metalloproteinases, which directly damage nerve cells (McGrath et al., 2015; Wilson et al., 2017).

Macrophages that bind to CD169 can secrete a large number of cytokines while, presenting antigens, particularly type I IFN, increasing the cascade reaction produced by cytokines, leading to the influx of DCs, neutrophils and NK cells (Grabowska et al., 2018; Herzog et al., 2022). In addition, CD169 + macrophages may also present lipid antigens to promote the activation of invariant natural killer T (iNKT) cells by expressing the MHC class of molecules, and iNKT cells further exacerbate damage to nerve cells by secreting cytokines to activate DC, NK, B and T cells (Covarrubias et al., 2014). On the other hand, Macrophages usually maintain immune homeostasis by phagocytosis of foreign particles and production of anti-inflammatory factors, such as IL-10 (Shapouri-Moghaddam et al., 2018). Both pro-inflammatory cytokines (IL-6, TNF-α, IFN-*γ*) and anti-inflammatory cytokines (IL-10) exhibit elevated levels, indicating a complex immune imbalance in which Siglec-1 may play a dual role. Siglec-1 might not only promote macrophage activation, thereby exacerbating detrimental inflammation, but also influence anti-inflammatory responses aimed at limiting tissue damage. However, it is noteworthy that the sources of these elevated cytokines remain unknown. Macrophages may be one of the potential sources, and this requires further clarification in subsequent studies focusing on target gene and cellular regulation. As an immune checkpoint, Siglec-1 may finely modulate the equilibrium between inflammation and its resolution. Dysregulation of Siglec-1 could potentially accelerate age-related hearing loss by amplifying neuroinflammation and diminishing neuronal resilience. Siglec-1 (CD169/sialoadhesin) is a macrophage-restricted adhesion receptor that lacks classical ITIM or ITAM signaling motifs but modulates inflammation through cytokine regulation and cell–cell interactions (Crocker et al., 1994). Unlike most Siglec family members that transduce inhibitory signals via ITIM motifs, Siglec-1 functions primarily as a co-receptor that fine-tunes macrophage activation states rather than simply suppressing them. Siglec-1 promotes the secretion of pro-inflammatory chemokines including MIP-1*α*, MIP-1*β*, MCP-1, and MIP-2, thereby amplifying local inflammation and recruiting additional immune cells. In coronary artery disease patients, Siglec-1 expression on monocytes correlates positively with hs-CRP (*r* = 0.316, *p* = 0.016) and Gensini score (*r* = 0.338, *p* = 0.015), supporting its role as a driver of inflammatory pathology (Xiong et al., 2009). Conversely, Siglec-1 also participates in anti-inflammatory responses. In a neurodegeneration model (ceroid lipofuscinosis, CLN), Siglec-1-deficient mice exhibit significantly lower pro-inflammatory cytokines (IL-1β, TNF-α) and higher anti-inflammatory cytokines (TGF-β), along with reduced axonal degeneration and extended lifespan. This indicates that Siglec-1 can sustain a detrimental inflammatory state, and its absence shifts the balance toward resolution (Groh et al., 2016). Thus, Siglec-1 appears to function as a rheostat—its engagement promotes inflammation, while its modulation permits resolution.

Macrophages are key players in the cochlear immune environment, particularly in the spiral ganglion, contributing to both normal function and ARHL pathology. Previous studies have revealed that the distribution and function of cochlear-resident macrophages are significantly specific. They exist under steady-state conditions and can be activated and recruit new macrophages when tissue damage occurs (York et al., 2007; Zhang, Roth et al., 2016b; Frye et al., 2017; Dong et al., 2018; van Dinther et al., 2018; Li et al., 2023). Unlike M1 and M2 macrophages, CD169 + macrophages can directly interact with T cells, B cells and dendritic cells (DCs) via CD169, thereby participating in immune regulation. Gangliosides, which are found in a cell-specific distribution in human cells and tissues, are ligands for Siglec-1 in macrophages and can drive Siglec-1-mediated signaling (Chang and Nizet, 2020; Raïch-Regué et al., 2023; Schnaar, 2023). Cells expressing CD169 have a high affinity for α2-3-glycosyltransferase and glucosidase, and communicate with other immune cells by recognizing and binding to polysaccharides on the surface of other immune cells, such as CD43 on T cells (Komohara et al., 2017; Edgar et al., 2019). Although the specific mechanism by which it causes an imbalance of inflammatory factors through macrophages remains unclear, we can still draw inspiration from the molecular mechanisms of various diseases involving CD169 + macrophages, such as autoimmune diseases, colitis, coronary atherosclerosis, and cancer (Bao et al., 2010; Asano et al., 2011, 2015; Zhang, Li et al., 2016a; Wang et al., 2017; Liu et al., 2021; Park et al., 2022; Li et al., 2024; Qi et al., 2024; Fukuda et al., 2025). Our research has revealed a notable elevation in Siglec-1 levels in aged mice, which is closely associated with increased macrophage activation (marked by F4/80 and MHC-II) and heightened levels of inflammatory cytokines. Macrophage activation was predominantly of the M1 phenotype, while the number of M2 macrophages did not change appreciably. That said, the possibility that individual M2 macrophages exhibited enhanced functional activity cannot be entirely excluded. M1 macrophages are primarily involved in pro-inflammatory responses, characterized by the upregulated secretion of pro-inflammatory cytokines by both M1 macrophages themselves and the associated immune cells. In contrast, M2 macrophages are mainly implicated in anti-inflammatory processes, such as tissue repair and remodeling. Siglec-1 may potentially enhance macrophage antigen presentation and modulate inflammatory responses. In ARHL, Siglec-1-positive macrophages sustain a chronic inflammation with imbalanced pro- and anti-inflammatory cytokines, influencing macrophage polarization and worsening spiral ganglion neuron damage. Targeting Siglec-1 may adjust macrophage activity, restore immune balance, reduce inflammation, and slow neuronal loss. Thus, Siglec-1 in cochlear macrophages is a promising target for immunomodulatory therapies to preserve hearing.

The positive correlation between Siglec-1 mRNA levels in peripheral blood and hearing loss severity in elderly patients highlights Siglec-1’s promise as a non-invasive biomarker for ARHL. Elevated Siglec-1 reflects systemic immune-inflammatory changes linked to SGN degeneration. As a liquid biopsy marker, it enables early detection before significant neuronal loss, supporting timely and personalized intervention. Monitoring blood Siglec-1 is practical for tracking ARHL progression and treatment response, suitable for routine screening in aging populations due to easy sampling. Further validation is required before clinical implementation.

Future research should focus on clarifying Siglec-1’s molecular mechanisms in regulating SGN macrophage signaling, identifying downstream effectors and interactions in ARHL-related inflammation and macrophage activation. Rigorous, large-scale clinical studies are essential to validate Siglec-1 as a reliable early diagnostic biomarker for ARHL, assessing its sensitivity, specificity, and prognostic value.

This study has limitations and methodological considerations for contextualizing findings and guiding future research. First, the small sample size in transcriptomic and clinical analyses may limit the generalizability and statistical robustness of correlations between Siglec-1 expression and ARHL severity, so larger, more diverse cohorts are needed. Second, species-specific differences between C57BL/6 J mice and humans must be considered, necessitating complementary studies utilizing human tissues or alternative models. Third, the complexity of immune-inflammatory processes within the aging SGN hinders the delineation of mechanistic pathways, highlighting the necessity of multi-omics approaches are essential. Furthermore, additional regulatory experiments (e.g., knockdown or overexpression) are warranted to functionally validate the direct effects of Siglec-1 on macrophages. Moreover, another limitation of the study lies in the comparison between elderly individuals with hearing loss and young, healthy individuals. This approach makes it impossible to exclude the influence of other age-related non-auditory factors and thus fails to establish a direct link with hearing degeneration. However, due to the scarcity of individuals aged over 65 with absolutely normal hearing, we were unable to collect relevant data during the study period. To compensate for this shortcoming, we conducted a correlation analysis between different degrees of hearing impairment and the content of Siglec-1, aiming to strengthen the direct association. Going forward, we will continue to monitor and collect blood information from individuals aged over 65 with normal hearing. Additionally, from an ethical standpoint, obtaining human cochlear tissue specimens is not feasible. Although the level of Siglec-1 in the blood cannot directly mirror the immune processes occurring in the cochlear spiral ganglion, when considered alongside the correlation analysis between hearing function and Siglec-1 levels, it can still offer indirect insights into certain aspects of their relationship. Finally, methodological improvements will enhance Siglec-1’s reliability as a diagnostic and therapeutic target. Addressing these limitations will strengthen mechanistic understanding and facilitate effective ARHL interventions.

## Data Availability

The original contributions presented in the study are included in the article/supplementary material, further inquiries can be directed to the corresponding author/s.
